# TRPV1 and TRPV1-Expressing Nociceptors Mediate Orofacial Pain Behaviors in a Mouse Model of Orthodontic Tooth Movement

**DOI:** 10.3389/fphys.2019.01207

**Published:** 2019-09-20

**Authors:** Sheng Wang, Martin Kim, Zayd Ali, Katherine Ong, Eung-Kwon Pae, Man-Kyo Chung

**Affiliations:** ^1^Program in Neuroscience, Center to Advance Chronic Pain Research, Department of Neural and Pain Sciences, School of Dentistry, University of Maryland, Baltimore, MD, United States; ^2^Department of Orthodontic and Pediatric Dentistry, School of Dentistry, University of Maryland, Baltimore, MD, United States

**Keywords:** orthodontic tooth movement, trigeminal ganglia, TRPV1, peptidergic nociceptors, periodontium, behavioral assays

## Abstract

Orthodontic force produces mechanical irritation and inflammation in the periodontium, which is inevitably accompanied by pain. Despite its prevalence, treatment of orthodontic pain is ineffective. Elucidating underlying neural mechanisms is critical to improving the management of orthodontic pain. We have assessed the contribution of transient receptor potential vanilloid subtype 1 (TRPV1) and the TRPV1-expressing subset of nociceptive afferents to pain behaviors induced by orthodontic force in mice. Microfocus X-ray computed tomography analysis showed that application of an orthodontic force of 10 g to the maxillary first molar produced reliable tooth movement in mice. Mouse grimace scale (MGS) was evaluated as an indication of non-evoked spontaneous pain and bite force (BF) was measured for assessing bite-evoked nocifensive behaviors. Orthodontic force increased MGS and decreased BF, both of which were interpreted as increased levels of pain. These behaviors peaked at 1d and returned near to the sham level at 7d. Retrograde labeling and immunohistochemical assays showed TRPV1-expressing peptidergic afferents are abundantly projected to the periodontium. Direct injection of resiniferatoxin into trigeminal ganglia (TG) decreased TRPV1-expressing afferents by half in the targeted region of TG. The chemical ablation of TRPV1-expressing afferents significantly attenuated orthodontic pain behaviors assessed by MGS and BF. Consistently, the knockout of TRPV1 also attenuated orthodontic force-induced changes in MGS and BF. These results suggest that TRPV1 and TRPV1-expressing trigeminal nociceptors constitute a primary pathway mediating orthodontic pain behaviors in mice. This model will be useful for mechanistic studies on orthodontic pain aimed at developing novel approaches for painless orthodontics.

## Introduction

Pain and discomfort are the major side effects of orthodontic treatment. Fixed orthodontic appliances produce pain in 94% of patients (Scheurer et al., 1996). Pain and soreness induced by orthodontic adjustment peaks after 24 h, and gradually declines, and resolution occurs within a week (Ngan et al., 1989; Scheurer et al., 1996). Pain during biting and chewing also peaks after 24 h, creating a major functional discomfort in daily life (Scheurer et al., 1996). Pain management during orthodontic treatment is often not effective, significantly affecting the patient’s compliance to treatment (Sergl et al., 1998). Peripheral and central mechanisms of orthodontic pain are under active investigation (Long et al., 2016) and better understanding of neurobiological mechanisms should help to better manage orthodontic pain.

Orthodontic force induces a variety of morphological and neurochemical responses of the peripheral and central nervous system (Long et al., 2016; Kobayashi and Horinuki, 2017). For mechanistic determination of orthodontic pain, it is critical to elucidate causal contributions of neural components in animal models involving pain behaviors. In rats, placement of a coil spring between the maxillary first molar and incisors produces changes in grimace scale and facial grooming (Yang et al., 2009; Liao et al., 2014). These behaviors are inhibited by morphine (Liao et al., 2014; Gao et al., 2016), indicating that these behaviors are relevant to nociception. A recent study also suggested that measuring changes in biting force in rats provides a functional surrogate outcome for biting-induced pain by orthodontic force (Long et al., 2019). However, similar models for assessing orthodontic pain behaviors have not been well established in mice, precluding the use of various genetic tools available in mice for mechanistic study of orthodontic pain.

The peripheral and central ascending pain pathways and neuronal circuitry that mediate orthodontic pain are not well defined. Orthodontic force should lead to the activation of periodontal nociceptors. However, the identity of the nociceptor subpopulation responsible for orthodontic pain is not well known. Periodontal ligament (PDL) contains Aδ and C nociceptive terminals (Byers, 1985). One of the well-defined populations of nociceptors is a peptidergic nociceptor containing the neuropeptides such as calcitonin gene-related peptide (CGRP). Peptidergic nociceptors project into the PDL in mice (Sarram et al., 1997). However, the role of periodontal peptidergic afferents in spontaneous pain or function-related pain evoked by orthodontic force is unknown.

Peptidergic afferents are enriched with transient receptor potential vanilloid subtype 1 (TRPV1), a receptor for capsaicin and noxious heat (Caterina et al., 2000; Cavanaugh et al., 2011). Although TRPV1 contributes to thermal hyperalgesia in skin (Caterina et al., 2000), TRPV1 mediates spontaneous pain and mechanical hyperalgesia during orofacial muscle inflammation (Chung et al., 2016; Wang et al., 2017a). The role of TRPV1 in non-evoked pain upon the application of orthodontic force was also suggested. Pharmacological inhibition or knockdown of TRPV1 attenuates facial grooming or grimace scale induced by orthodontic force in rats (Gao et al., 2016; Guo et al., 2019a). However, it is not known if TRPV1 contributes to other modalities of orthodontic pain, such as bite-evoked pain. Given the differential contribution of TRPV1 to spontaneous pain and bite-evoked pain under muscle inflammation (Wang et al., 2017a), it is important to determine the role of TRPV1 in bite-evoked nocifensive behaviors during orthodontic tooth movement.

In this study, we have determined the contribution of TRPV1 and TRPV1-expressing afferents to orthodontic pain behaviors in a mouse model of orthodontic tooth movement. We have assessed a mouse model of orthodontic pain using two different behavioral measurements. In combination with targeted chemical ablation of specific neuronal subtypes and genetic inhibition, we tested the hypothesis that TRPV1 and TRPV1-expressing trigeminal nociceptors constitute a major pathway for transduction of orthodontic pain.

## Materials and Methods

### Experimental Animals

C57BL/6 mice (The Jackson Laboratory, Bar Harbor, Maine), TRPV1 KO mice (Caterina et al., 2000), TRPV1-Cre mice (Jax #017769) (Cavanaugh et al., 2011), and Rosa26-mT/mG (Jax #007576) (Muzumdar et al., 2007; Wang et al., 2017b) were used. In experiments involving behavioral assays, 12-week-old mice were used for stable measurement of bite force (BF) behaviors (Wang et al., 2017a). Orthodontic pain shows limited or no clear sex difference (Jones, 1984; Ngan et al., 1989; Scheurer et al., 1996), whereas mouse behavioral assays, especially BF measurement, are influenced by sex; when both male and female mice are included in pain assays, variation of the data increases (Guo et al., 2019b). To facilitate establishing a model of orthodontic pain in mice, we have focused on male mice without attempting to determine sex differences at this time. All animal procedures were consistent with the NIH Guide for the Care and Use of Laboratory Animals (Publication 85-23, Revised 1996), and were performed according to a University of Maryland-approved Institutional Animal Care and Use Committee protocol.

### Experimental Orthodontic Tooth Movement

**Figure 1:**
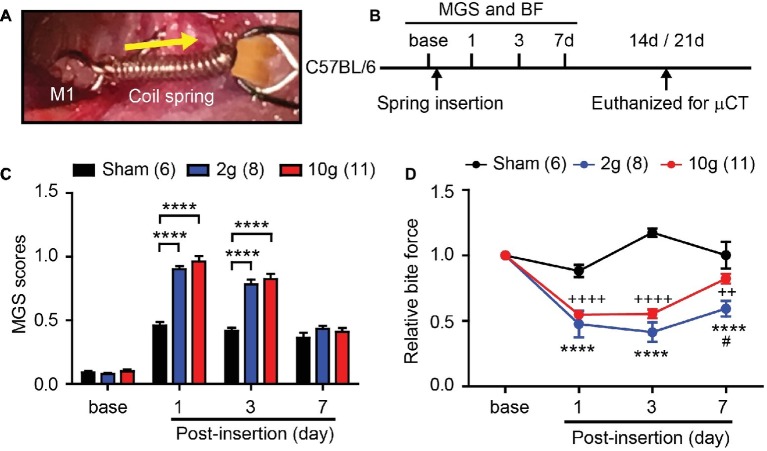
Orthodontic force induces spontaneous and bite-evoked pain behaviors in mice. **(A)** Ni-Ti closed coil spring was placed between the left maxillary first molar (M1) and incisors (INC). The spring was activated to exert 2 or 10 g force in the orthodontic force group. In the sham group, the spring did not produce active force. Arrow, direction of orthodontic force. **(B)** Timeline of experiment. In adult C57BL/6 mice, behavioral assays were performed before and after the placement of an orthodontic spring. BF assay was performed immediately after video recording for facial grimace assay on each day. The mice were euthanized for μCT at 14d, with the exception that five mice in the 2 g group and all mice in the sham group were euthanized at 21d. BF, bite force assay; MGS, mouse grimace scale; μCT, micro-CT. **(C)** Comparison of MGS scores at different time points. Numbers within parentheses represent number of animals. ****p < 0.0001 in two-way repeated-measure (RM) ANOVA followed by Bonferroni *post hoc* test. **(D)** Comparison of relative BF at different time points. ****p < 0.0001 (2 g vs. sham), ^++^*p* < 0.01, ^++++^*p* < 0.001 (10 g vs. sham), ^#^*p* < 0.05 (2 g vs. 10 g) in Bonferroni *post hoc* test following two-way RM ANOVA.

To produce orthodontic forces in mice, a coil spring was placed between maxillary first molar and maxillary incisors (Figure 1A). The animals were anesthetized with ketamine (100–150 mg/kg) and xylazine (10–16 mg/kg). A 0.010-in stainless steel ligature wire was looped around the first molar, and a second ligature wire was looped around maxillary incisors. We used two nickel-titanium orthodontic coil springs (Xu Jia Chuang Spring; Guangdong, China) exerting different forces: a 2 g spring (wire diameter: 0.1 mm; outer diameter: 1.6 mm; length: 1.8 mm) exerts 2 ± 0.2 g force, whereas a 10 g spring (wire diameter: 0.15 mm; outer diameter: 1.8 mm; length: 2.2 mm) exerts 10 ± 1 g force upon activation of 1 mm. In the group with orthodontic force (OF), the coil spring was extended mesially and ligated to the incisors. In the sham group, the orthodontic spring was irreversibly deformed by extension beyond elastic limit and ligated so that the spring delivered no force. To secure the ligature wires, self-etching primer and light-cured adhesive resin cement (Transbond; 3 M Unitek, Monrovia, California) were applied to the palatal surfaces of the maxillary incisors and first molars. After spring insertion, the animals were supplied with soft diet (Dietgel recovery; ClearH2O; Portland, ME). The appliances were inspected daily, and additional bonding material was applied as necessary.

### Microfocus Computed Tomography

The animals were anesthetized by ketamine/xylazine, and euthanized by transcardial perfusion using 3.7% paraformaldehyde. Maxillae were hemisected, and microfocus computed tomography (μCT) images were obtained using a Siemens Inveon Micro-PET/SPECT/CT (Siemens, Ann Arbor, MI) with 9 μm spatial resolution. Siemens Inveon Research Workplace 4.2 software was used for image acquisition and processing, 2-D and 3-D image viewing, and quantitative analysis. The intermolar distance was measured as the distance between the most distal point of the maxillary first molar crown and the most mesial point of the maxillary second molar crown. The measurements were performed in the sagittal plane because this plane showed the most root structure, permitting estimation of angulation of the long axis of the tooth. The two-dimensional images were magnified 10 times for more precise line drawings at the closest proximity of the two convex molar crown surfaces. Bone volume fraction in the furcation region of the maxillary first molar was assessed as a quantitative analysis of alveolar bone changes. The region of interest for the total interradicular alveolar bone space [tissue volume (TV)] in the furcation region was defined as previously described (Yadav et al., 2015). Within the region of interest, total amount of actual interradicular alveolar bone volume (BV) was calculated, which was divided by TV to calculate bone volume fraction.

### Microinjection Into Trigeminal Ganglia

To selectively ablate TRPV1-expressing trigeminal nociceptors, resiniferatoxin (RTX) was directly injected into trigeminal ganglia (TG). RTX is a highly efficacious agonist of TRPV1, and the activation of TRPV1 by RTX leads to ablation of nociceptor terminals or soma upon localized injection (Karai et al., 2004; Chung and Campbell, 2016). The animals were anesthetized using ketamine/xylazine and placed in a Kopf stereotaxic apparatus. A midline incision of 3–5 mm and an opening to the skull were made. A 0.5-μl Hamilton micro-syringe was used for microinjection. The micro-syringe needle was placed in the left TG regions according to the stereotaxic coordinates of the mouse brain (0.2 mm posterior to bregma, 1.3 mm lateral to the midline, and 6.5 mm deep) for targeting ophthalmic/maxillary (V1/V2) region. RTX (50 ng/0.5 μl; Sigma-Aldrich) was dissolved in phosphate buffered saline (PBS) containing 1% dimethyl sulfoxide and 10% Tween-80. Mice injected with vehicle (0.5 μl) served as a control group. Injection was performed at a rate of 0.5 μl/min and the injection needle was held in the tissue for 2 min to allow diffusion before removal.

### Measurement of Mouse Grimace Scale and Bite Force Measurement

Mouse grimace scale (MGS) and BF were performed as previously described (Wang et al., 2017a, 2018; Guo et al., 2019b). All behavioral assays and video analysis were performed in a blinded manner. For MGS assay, the mice were videotaped for 30 min in each experimental time point and 10 images per 30 min session were manually captured. The scores of the five action units in each photograph were averaged, and a mean MGS score was obtained from the 10 images, which was presumed to reflect the level of spontaneous pain. For BF assay, mice were placed in a modified 60-ml plastic syringe with a wide opening at one end to accommodate the head of the mouse. To minimize stress, the mouse was released immediately from the syringe if it vigorously moved or tried to hide inside the syringe. The syringe containing the mouse was held manually and moved slowly at 0.5–1 cm/s toward bite plates so that the mouse could bite the plates. Spike 2 software was used to measure the voltage changes from transducer displacement. SigmaPlot 8.0 was used to convert the voltage change into force based on calibration using standard weights. Bite force was recorded for 120 s per session and the top five force measurements were averaged.

### Measurement of Eye-Wiping Behavior

To functionally verify the effective ablation of TRPV1-expressing afferents by intra-TG injection of RTX, we performed an eye-wiping test using capsaicin. The animals were placed in a plastic container (9 cm × 9 cm × 13 cm) with two-mirrored back walls to allow the video camera to record a four-sided view. Two drops (20 μl) of 0.03% capsaicin solution were placed onto the left conjunctiva of the eye. The number of eye wipes with the ipsilateral forepaw in a 5-min window was counted.

### Retrograde Labeling of Periodontal Afferents

In C57BL/6 mice anesthetized by Ketamine/Xylazine, fluorogold (FG; Fluorochrome) was injected into gingiva around maxillary first molar to retrogradely label periodontal afferents in TG. FG was dissolved in 0.9% saline at a concentration of 4%. A 50-μl Hamilton syringe was used to slowly inject 5 μl of tracer into five sites (1 μl per site) at gingiva around disto-buccal groove, buccal groove, mesial groove, palatal groove, and disto-palatal groove of the maxillary first molar. The mice were euthanized 7 days following the injection by transcardial perfusion for further histological study. Four ganglia were analyzed for quantification.

### Immunohistochemistry of Trigeminal Ganglia and Maxillae

Immunohistochemical assays of TG and maxillae were performed as previously described (Chung et al., 2011, 2012; Wang et al., 2017a). Maxillae were decalcified in 10% EDTA (pH 7.4) for 7 days at 4°C. Tissues were cryoprotected and cryosectioned at 12 μm for TG and 30 μm for decalcified maxillae. Conventional immunohistochemical procedures were performed with rabbit anti-TRPV1 (1:1,000; a generous gift from Dr. Michael Caterina at Johns Hopkins University), guinea pig anti-CGRP (1:1,000; Penninsula Labs), or rabbit green fluorescent protein (GFP; 1:1,000, Invitrogen). We verified the specificity of the primary antibodies by using genetically engineered mice lacking the expression of the target gene or by omitting the primary antibody (Chung et al., 2012). The sections were further incubated with appropriate secondary antibodies (Invitrogen). Tooth sections were stained with 4′,6-diamidino-2-phenylindole (DAPI) to visualize the cellular nuclei. For classification of neuronal size in TG sections, we measured the cross-sectional area of the neurons in ImageJ and followed the criteria described elsewhere (small, < 300 μm^2^; medium, 300–600 μm^2^; large, > 600 μm^2^) (Ichikawa et al., 2006). For counting TG neurons, Nissl staining was performed using NeuroTrace 500/525 green fluorescent Nissl Stain (Invitrogen). Four to five images from V1/V2 regions and three to four images from mandibular (V3) regions were taken from each TG.

### Statistical Analysis

Data are presented as mean ± standard error of the mean. Statistical comparisons were performed using Student’s *t*-test or analysis of variance (ANOVA) followed by Bonferroni *post hoc* test as indicated in figure legends. The criterion for statistical significance was *p* < 0.05. All statistical analyses were performed using Prism (GraphPad Software, La Jolla, CA).

## Results

### A 10 g Orthodontic Force Reliably Produces Pain and Tooth Movement in Mice

In mice, the range of orthodontic force used for producing tooth movement is between 3 and 50 g (Yan et al., 2015; Rangiani et al., 2016; Yadav et al., 2016; Liu et al., 2017; Odagaki et al., 2018). Using two coil springs exerting either 2 or 10 g (Figure 1A), we performed an experiment (Figure 1B) to determine the amount of orthodontic force required to effectively produce pain and tooth movement. Mouse grimace scale (MGS) scores among three groups (sham, 2 g, and 10 g) showed significant difference over 7 days following procedure (Figure 1C; interaction of time and group effect, F_6,66_ = 14.61, *p* < 0.0001). At baseline, there were no differences in MGS scores between groups (Figure 1C). At 1d and 3d after spring insertion, MGS scores of 2 and 10 g groups were similar and significantly higher than those of the sham group. At 7d, there were no differences in MGS scores among the three groups. Changes in bite force (BF) were also analyzed (Figure 1D) and three groups showed significant difference over 7 days following procedure (Figure 1D; interaction of time and group effect, F_6,60_ = 15.67, *p* < 0.0001). At 1d and 3d, BF of 2 and 10 g groups was significantly reduced compared to the sham group. At 7d, BF had partially recovered toward the baseline in both 2 and 10 g groups, but remained significantly lower than that of the sham group. Interestingly, the recovery in the 2 g group was slower than in the 10 g group, such that at 7d, BF was significantly lower in the 2 g group than in the 10 g group.

**Figure 2:**
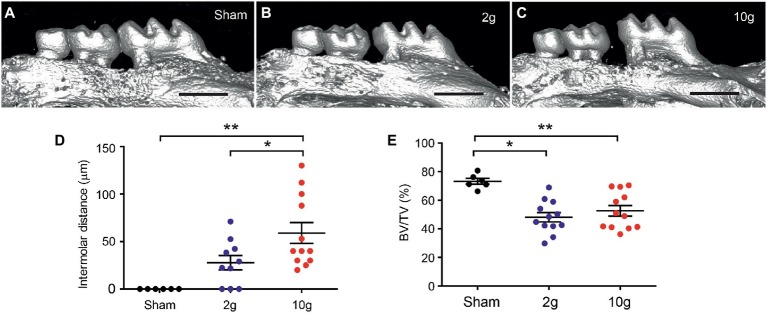
Orthodontic force of 10 g produces reliable tooth movement in mice. **(A–C)** Examples of 3D constructed μCT images of sham **(A)**, 2 g **(B)**, and 10 g **(C)** force groups. Intermolar distance was measured as 49 and 86 μm in **(B** and **C)**, respectively. Scale bar, 1 mm. *p < 0.05, **p < 0.005 in Holm-Sidak’s multiple comparison post-test following one-way ANOVA. **(D,E)** Comparison of intermolar distances **(D)** and bone volume fraction **(E)**. BV/TV, bone volume/tissue volume; *p < 0.05, **p < 0.005 in Holm-Sidak’s multiple comparison post-test following one-way ANOVA.

All mice in the 10 g group and five mice in 2 g group were euthanized 14d after spring insertion. All mice in the sham group and five mice in the 2 g group were euthanized 21d after spring insertion. Micro-CT analysis was performed to evaluate the extent of tooth movement (Figure 2). The sham group did not produce tooth movement during the 3 weeks (Figures 2A,D). The 2 g spring produced 25.1 ± 8.4 μm (n = 5) of mesial movement of the first molar during 2 weeks, which was not significantly different from the amount during 3 weeks (30.4 ± 13.6 μm; *n* = 5; *p* > 0.7; Student’s *t*-test). When, the 2 g data from the two time points were pooled, the three groups (sham, 2 g, and 10 g) showed significant difference in tooth movement (F_2,27_ = 10.6, *p* = 0.0004). Intermolar distances in 2 g group were not significantly different from sham (*p* = 0.076; Figure 2D). Among 10 samples in the 2 g group, three showed no tooth movement. Although 3 g force produces tooth movement well in juvenile 5-week-old mice (Rangiani et al., 2016), 2 g force was not as effective in our 12-week-old mice. In contrast, the 10 g spring produced 59 μm of tooth movement after 2 weeks, which was significantly different from sham or 2 g groups (Figures 2C,D; *p* = 0.0013 vs. sham; *p* = 0.037 vs. 2 g). In comparison of bone volume fraction, the three groups showed significant difference (Figure 2E; F_2,25_ = 8.78, p = 0.0013). Both 2 and 10 g springs produced significantly reduced bone volume fraction (BV/TV) compared to sham, which indicates active bone remodeling has occurred in both groups. However, there was no difference between 2 and 10 g groups. Based on these results, we regarded 10 g as the force of choice to reliably produce tooth movement during a 2-week period, and 10 g was used in the remainder of studies.

### Periodontium Is Innervated by TRPV1-Expressing Peptidergic Afferents

**Figure 3:**
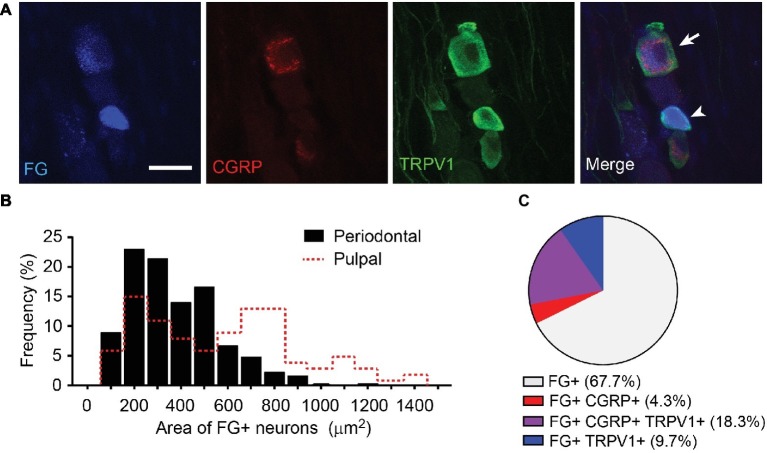
Neurochemical properties of retrogradely labeled periodontal afferents in mice. **(A)** Representative images of periodontal afferents retrogradely labeled using fluorogold (FG) co-labeled with TRPV1 and CGRP. Arrowhead, FG+ TRPV1+ neuron; arrow, FG+ TRPV1+ CGRP+ neuron; scale bar, 30 μm. **(B)** Distribution of cross-sectional area of FG-labeled periodontal afferents (*n* = 313). Size distribution of FG+ pulpal afferents in mice (red dotted line) was derived from published data (Chung et al., 2011) for comparison. **(C)** Proportion of TRPV1+ and CGRP+ afferents among FG-labeled periodontal afferents. A total of 313 FG+ neurons were analyzed.

To determine the major primary afferents subpopulation transducing orthodontic pain, we determined neurochemical properties of primary afferents projected to periodontium (Figure 3). We injected the retrograde labeling tracer fluorogold (FG) into gingiva around the maxillary first molars in mice. FG-labeled TG neurons showed various sizes and neurochemical properties (Figure 3A). Among 313 FG-labeled neurons, small-, medium-, and large-sized afferents compose 44, 44, and 12%, respectively (372 ± 11 μm^2^; Figure 3B). When these data were compared with published data from FG-labeled pulpal afferents (Chung et al., 2011), the size of periodontal afferents was significantly smaller than pulpal afferents (586 ± 33 μm^2^; *n* = 99; *p* < 0.0001; Figure 3B, red dotted line). When the extent of co-localization of FG, CGRP, and TRPV1 was determined (Figure 3C), 23% of the periodontal afferents expressed CGRP and 28% of periodontal afferents expressed TRPV1. Eighty-one percent of CGRP-expressing periodontal afferents were co-expressed with TRPV1.

**Figure 4:**
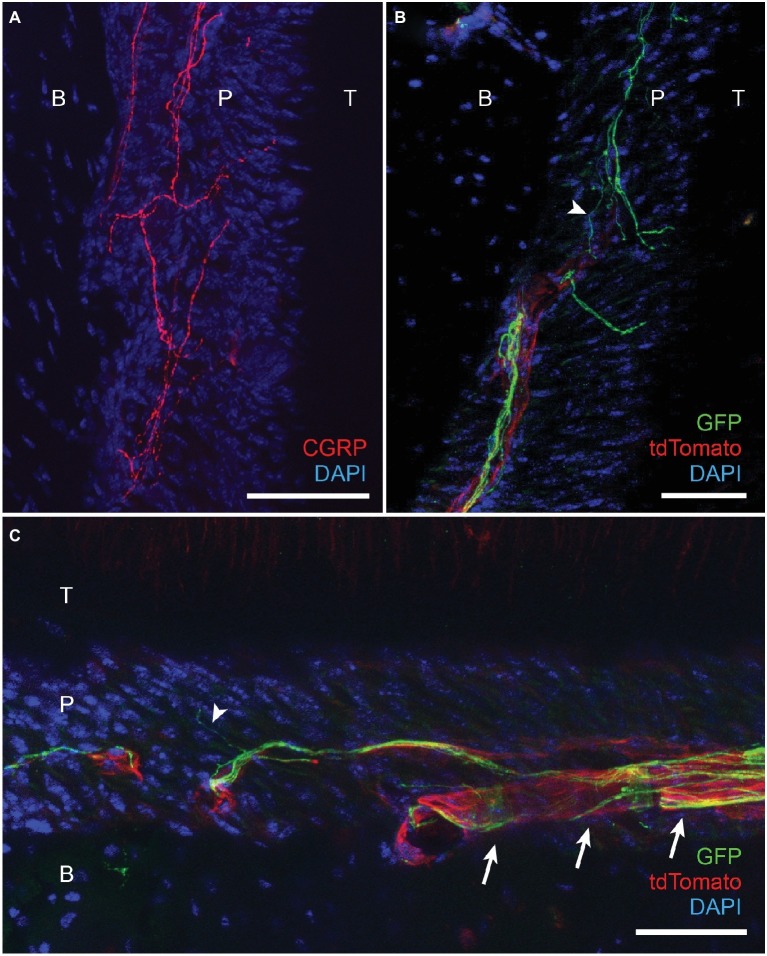
Projection of CGRP-expressing and TRPV1-lineage afferents to periodontal ligaments in mice. **(A)** Immunohistochemical labeling of CGRP in periodontium from C57BL/6 mice. T, tooth; P, periodontal ligament; B, alveolar bone; scale bar, 30 μm. **(B)** Immunohistochemical labeling of GFP in TRPV1-GFP mice (TRPV1-Cre X Rosa26-mT/mG) labeling TRPV1-lineage neurons. B, bone; P, PDL; T, tooth; scale bar, 50 μm. In this mouse line, tdTomato labels all neuronal and non-neuronal cells except TRPV1-lineage afferents. Arrowhead, an example of fine GFP-expressing terminal. **(C)** Immunohistochemical labeling of GFP in TRPV1-GFP mice labeling TRPV1-lineage neurons. Scale bar, 50 μm; arrowhead, an example of fine GFP-expressing terminal; arrows, examples of GFP-expressing nerve terminals associated with blood vessel.

We further determined the neurochemical properties of nerve terminals within PDL by immunohistochemical labeling of afferent terminals in decalcified periodontal tissues (Figure 4). CGRP-expressing terminals were densely projected into the PDL (Figure 4A), which is consistent with previous reports (Sarram et al., 1997). We also attempted immunohistochemical labeling of TRPV1-expressing nerve terminals in decalcified periodontium. However, we were not able to observe convincing TRPV1 labeling of nerve terminals within PDL. As an indirect approach, we took advantage of TRPV1-Cre mice for GFP labeling of TRPV1-lineage neurons (Cavanaugh et al., 2011). In this mouse line, approximately half of GFP-expressing neurons express TRPV1 in ganglia (Cavanaugh et al., 2011). TRPV1-Cre line was crossed with R26-mT/mG line to express membrane-bound GFP from TRPV1-lineage afferents. To maximize labeling of nerve terminals, we performed immunohistochemical labeling of GFP using a specific antibody. In this line, tdTomato is also expressed from all neuronal and non-neuronal cells other than TRPV1-lineage afferents. Under this condition, GFP-expressing nerve terminals were clearly visible in the PDL (Figure 4B). GFP-expressing terminals were often observed in close proximity with blood vessels within the PDL (Arrows in Figure 4C). The thickness of the GFP-expressing axonal terminals was various and there was a subpopulation of fine terminals (arrowheads in Figures 4B,C) that are presumably unmyelinated C fibers. These results suggest that TRPV1-expressing peptidergic afferents constitute a major subset of afferents in the periodontium including PDL and likely mediate orthodontic pain.

### Ablation of TRPV1-Expressing Nociceptors Attenuates Orthodontic Pain Behaviors

**Figure 5:**
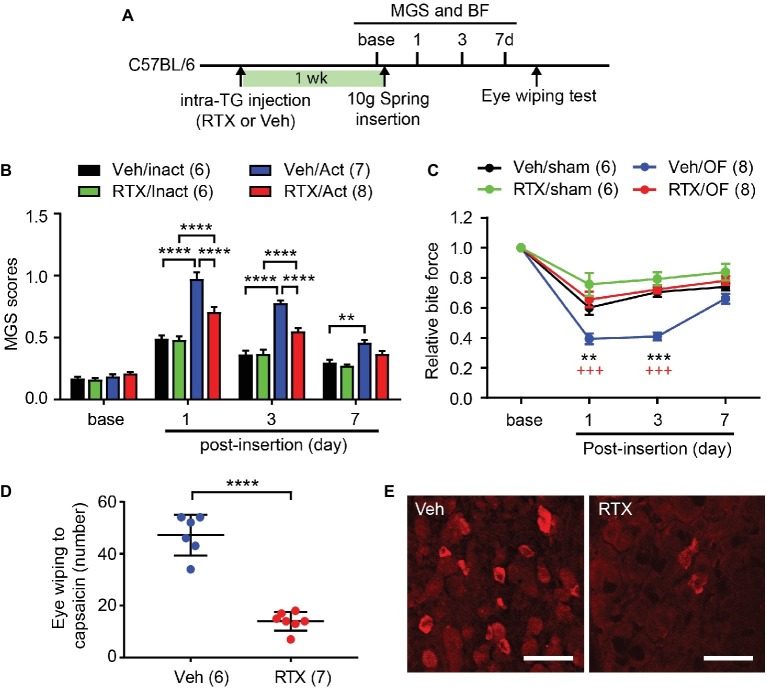
Chemical ablation of TRPV1-expressing trigeminal afferents attenuates orthodontic pain behaviors. **(A)** Timeline of experiment. Resiniferatoxin (RTX, 50 ng in 0.5 μl PBS) or vehicle (Veh) was stereotaxically injected into left trigeminal ganglia (TG) in adult C57BL/6 mice 1 week before the placement of the 10 g spring. **(B)** Comparison of MGS scores at different time points. Numbers within parentheses represent number of animals. **p < 0.01, ****p < 0.0001 in Bonferroni *post hoc* test following two-way RM ANOVA. OF, 10 g orthodontic force; RTX, resiniferatoxin; Veh, vehicle. **(C)** Comparison of relative BF at different time points. **p < 0.01, ***p < 0.0001 (vs. Veh/sham), ^+++^*p* < 0.001 (vs. RTX/OF) in Bonferroni *post hoc* test following two-way RM ANOVA. **(D)** Number of eye-wiping motions measured over 5 min following the application of capsaicin (0.03% in 20 μl) to the ipsilateral eye in mice with intra-TG injection of vehicle or RTX. ****p < 0.0001, Student’s *t*-test. **(E)** Immunohistochemical labeling of TRPV1 in TG from mice injected with Veh or RTX into TG. Scale bar, 50 μm.

For selective ablation of TRPV1-expressing nociceptors, we injected RTX to one side of TG (Figure 5A). After a week, behavioral assays were performed before and after insertion of the spring. MGS scores among groups showed significant difference over 7 days following procedure (Figure 5B; interaction of time and group effect, F_9,69_ = 16.1, *p* < 0.0001). There were no differences in MGS scores between groups at baseline (Figure 5B). At 1d, the group receiving vehicle and subjected to 10 g OF (Veh/OF) had significantly higher MGS scores than mice receiving vehicle and sham treatment (Veh/Sham). RTX-treated mice receiving OF (RTX/OF) also had increased MGS compared to sham (RTX/Sham). RTX/OF mice exhibited significantly lower MGS scores than Veh/OF mice. At 3d, the trend was similar to the 1d result. At 7d, the Veh/OF group but not the RTX/OF group showed significant differences in MGS compared to sham controls.

In the BF assay (Figure 5C), changes in bite force showed significant difference over 7 days following procedure (interaction of time and group effect, F_9,72_ = 7.814, *p* < 0.0001). The Veh/OF group showed a significant reduction in BF compared to the Veh/Sham group at 1d. In contrast, RTX-treated groups showed no significant differences in BF between OF and sham groups. BF reduction was significantly less in the RTX/OF group compared to the Veh/OF group at both 1d and 3d. At 3d, the Veh/sham group showed a recovery in BF to levels similar to RTX-treated groups, while Veh/OF group continued to exhibit a significant reduction in BF compared to the RTX/OF group. At 7d, there were no significant differences between BF among experimental groups.

After pain measurements were completed, the mice underwent the capsaicin eye-wiping test. Following application of capsaicin to the ipsilateral eye, RTX-treated mice exhibited significantly fewer eye wipes than controls (Figure 5D; t(11) = 10.08, *p* < 0.0001). In the post-mortem immunohistochemical staining, substantial reduction in TRPV1 expression was observed in RTX mice compared to controls (Figure 5E). These results validate ablation of trigeminal TRPV1-expressing afferents.

**Figure 6:**
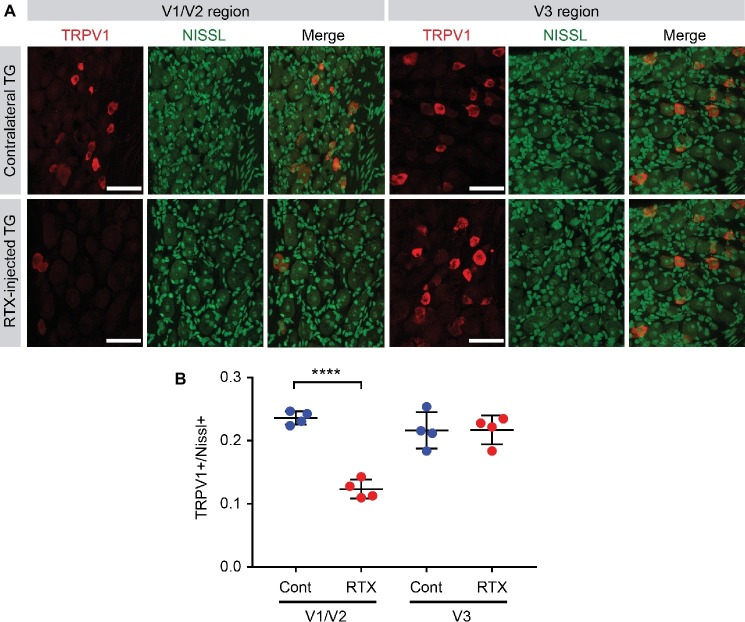
Efficacy of ablation of TRPV1-expressing nociceptors by intra-TG injection of RTX. **(A)** Immunohistochemical labeling of TRPV1 (red), Nissl staining (green), and merged images in ophthalmic/maxillary (V1/V2) area or mandibular (V3) area of TG from RTX-injected or uninjected contralateral side. **(B)** Proportion of TRPV1-expressing neurons among Nissl+ neurons. ****p < 0.0001 in Student’s *t*-test. *N* = 4 ganglia in each group.

To estimate the efficacy of ablating TRPV1-expressing afferents by intra-TG injection of RTX, we injected RTX into V1/V2 area of left side TG of four C57BL/6 mice (Figure 6). After a week, the mice were euthanized, and we compared the proportion of TRPV1-expressing afferents between RTX-injected TG and the uninjected contralateral TG (Figure 6A). To label all neurons, including ones not expressing TRPV1, Nissl staining was performed. As an internal control, we also compared TRPV1-expressing afferents in V3 region. Uninjected contralateral TG showed that TRPV1-expressing neurons account for 24% of all the Nissl-positive neurons in V1/V2 region and 22% in V3 region (Figures 6A,B). In contrast, the proportion of TRPV1-expressing neurons was reduced to approximately half (12%) in V1/V2 region of RTX-injected mice, which was significantly different from contralateral TG (Figure 6B; t(6) = 12, *p* < 0.0001). The proportion of TRPV1-expressing neurons in the untargeted V3 region of RTX-injected mice was 22%, which was not significantly different from V3 region of contralateral TG (*p* > 0.96). These results suggest that intra-TG injection of RTX produces ablation of approximately half of TRPV1-expressing neurons in V1/V2 region of TG.

### Genetic Inhibition of TRPV1 Attenuates Orthodontic Pain Behaviors

**Figure 7:**
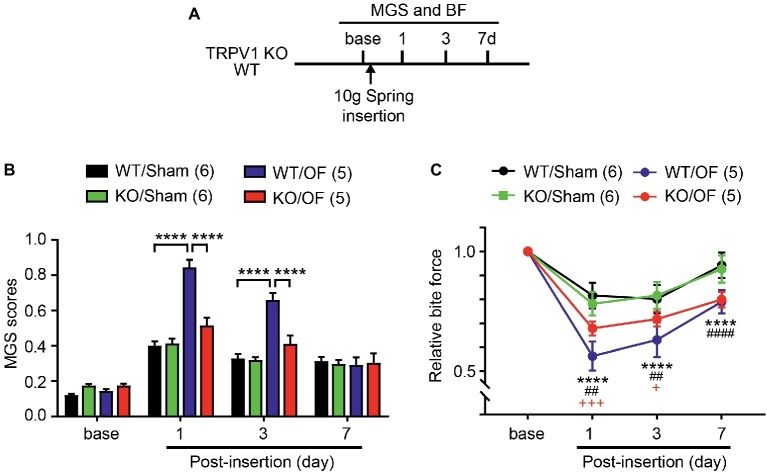
TRPV1 KO attenuates orthodontic pain behaviors. **(A)** Timeline of experiment. The 10 g spring was placed in TRPV1 KO and sham. **(B)** MGS before and 1, 3, and 7d after the application of 10 g orthodontic force (OF) or sham. ****p < 0.0001 in Bonferroni *post hoc* test following two-way RM ANOVA. **(C)** Relative BF from the same mice used for measuring MGS in A. ****p < 0.0001 (WT/sham vs. WT/OF); ^##^*p* < 0.01, ^####^*p* < 0.0001 (KO/Sham vs. KO/OF); ^+^*p* < 0.05, ^+++^*p* < 0.001 (WT/OF vs. KO/OF) in Bonferroni *post hoc* test following two-way RM ANOVA.

To determine the roles of TRPV1 in orthodontic pain, we evaluated MGS and BF in TRPV1 KO or WT littermates subjected to 10 g OF or sham (Figure 7A). MGS scores among groups showed significant difference over 7 days following procedure (Figure 7B; interaction of time and group effect, F_9,54_ = 15.38, *p* < 0.0001). There were no differences in MGS scores between groups at baseline. At 1d, WT mice subjected to 10 g OF (WT/OF) showed significantly higher MGS scores than WT mice receiving sham treatment (WT/Sham). In contrast, changes in MGS in TRPV1 KO mice receiving OF (KO/OF) did not show significant difference compared to sham (KO/Sham). Consequently, KO/OF mice exhibited significantly lower MGS scores than WT/OF mice. At 3d, the trend was similar to the 1d result. At 7d, the WT/OF group and the KO/OF group did not show significant differences in MGS compared to sham controls.

In the BF assay (Figure 7C), changes in bite force showed significant difference over 7 days following procedure (interaction of time and group effect, F_9,54_ = 9.882, *p* < 0.0001). The WT/OF group showed a significant reduction in BF compared to the WT/Sham group at 1d. KO/OF group also showed significantly reduced BF compared to KO/sham group. BF reduction in the KO/OF group was significantly less than reduction in the WT/OF group at both 1d and 3d. At 7d, BF of WT/OF and KO/OF had further recovered toward levels in sham groups. However, the BF of WT/OF and KO/OF were significantly lower than that of WT/sham and KO/sham groups, respectively. There were no significant differences between BF in WT/OF and KO/OF groups.

### Discussion

We have established a mouse model of orthodontic pain, and have tested two orthodontic force levels, 2 and 10 g. The 2 g force was great enough to produce comparable levels of pain as the 10 g force, but was not sufficient to produce consistent tooth movement. Therefore, we regarded 10 g as a minimum orthodontic force producing reliable tooth movement with maximal levels of pain in the mouse model. In clinic, orthodontic force induces spontaneous pain and chewing-evoked pain, which is resolved in approximately 1 week (Scheurer et al., 1996). Our mouse model reflects similar clinical characteristics: MGS and BF may reflect spontaneous pain and chewing-evoked pain, respectively, and orthodontic force-induced changes in MGS and BF lasted approximately a week. Thus, our mouse model mimics clinically relevant pain evoked by a reasonable force that produces orthodontic tooth movement.

We determined the neurochemical properties of afferents retrogradely labeled from periodontium including gingiva, alveolar bone, and periodontal ligament. The size distribution showed that majority of periodontal afferents are small to medium diameter. The size of periodontal afferents is apparently larger than facial skin afferents, similar to dural afferents but smaller than pulpal afferents (Chung et al., 2011; Huang et al., 2012). We found that 28% of TG afferents retrogradely labeled from mouse periodontium contained TRPV1 and 23% of periodontal afferents contained CGRP. The proportion of TRPV1-expressing periodontal afferents in mice is comparable to the proportion of TRPV1-expressing periodontal ligament afferents in rats (~25%) (Gibbs et al., 2011) and higher than in pulpal afferents in mice (~10%) (Chung et al., 2011). The proportion of CGRP-expressing afferents in mouse periodontal afferents is similar to the proportion in pulpal afferents (28%), lower than in mouse facial skin afferents (~30%) but higher than in dural afferents (~15%) (Chung et al., 2012; Huang et al., 2012). Importantly, the peptidergic periodontal afferents were highly colocalized with TRPV1. Therefore, chemical ablation of TRPV1-expressing afferents should affect a majority of peptidergic afferents that project to periodontium.

Using targeted chemical ablation, we found that TRPV1-expressing trigeminal nociceptors are major contributors to pain behaviors evoked by orthodontic force in mice. TRPV1-expressing afferents are responsible for thermal, but not mechanical, sensitivity in skin (Cavanaugh et al., 2009). In deep tissues, such as masseter muscle, however, TRPV1-expressing afferents mediate spontaneous pain as well as bite-evoked pain under inflammation (Wang et al., 2017a). Since orthodontic force induces inflammation in periodontium to produce inflammatory mediators and cytokines (Long et al., 2016; Kobayashi and Horinuki, 2017), it is likely that changes in MGS and BF involve peripheral sensitization of periodontal afferent terminals. Indeed, non-steroidal anti-inflammatory drugs (NSAIDs) reduce orthodontic pain in patients and rodents (Bartzela et al., 2009; Shibazaki et al., 2009). Pharmacological inhibition or knockdown of TRPV1 attenuates spontaneous pain behaviors, such as grimace scale or face grooming, induced by orthodontic forces in rats (Gao et al., 2016; Guo et al., 2019a). Our experiments using genetic knockout of TRPV1 further support the contribution of TRPV1 to spontaneous pain behaviors evoked by orthodontic force. We also showed that knockout of TRPV1 attenuated BF reduction evoked by orthodontic force. This is in contrast to the results from inflamed masseter muscle, in which TRPV1 substantially contributes to MGS whereas it only marginally affects BF (Wang et al., 2017a). These results suggest that inhibiting TRPV1 can affect different modalities of orthodontic pain and that the contribution of TRPV1 to bite-evoked pain is context-dependent. The source of such different contribution of TRPV1 to bite-evoked nocifensive behaviors following masseter inflammation versus orthodontic tooth movement is not clear. It is possible that the extent of injury produced by masseter inflammation is more extensive than orthodontic tooth movement and, therefore, involves greater peripheral and central components that are independent of TRPV1.

Despite the clear role of TRPV1 and TRPV1-expressing afferents in orthodontic pain behaviors, we do not exclude possible contributions of other molecules and neurochemically distinct subtypes of nociceptors. Partial attenuation of MGS and BF by the ablation of TRPV1-expressing afferents or knockout of TRPV1 supports this notion. It is highly likely that other TRP channels enriched in peptidergic afferents, for example TRPA1, play additional or overlapping roles in orthodontic pain behaviors as in the case of masseter hyperalgesia (Wang et al., 2018). These possibilities need to be determined in the future.

Determining the mechanisms of orthodontic pain addresses a critical clinical problem in orthodontics. A major concern in the field is that conventional analgesics such as NSAIDs adversely affect orthodontic tooth movement (Bartzela et al., 2009). Therefore, understanding mechanisms of orthodontic pain should help in the development of new approaches for attenuating pain without deleteriously affecting orthodontic tooth movement.

In conclusion, our data support the hypothesis that TRPV1 and TRPV1-expressing trigeminal nociceptors constitute a major pathway for transduction of orthodontic pain. This study established a new mouse model of orthodontic pain, well suited to mechanistic studies aimed at developing novel approaches for painless orthodontics.

## Data Availability Statement

All datasets generated for this study are included in the manuscript/supplementary files.

## Ethics Statement

The animal study was reviewed and approved by All animal procedures were consistent with the NIH Guide for the Care and Use of Laboratory Animals (Publication 85-23, Revised 1996), and were performed according to a University of Maryland-approved Institutional Animal Care and Use Committee protocol.

## Author Contributions

SW, E-KP, and M-KC designed the experiments. SW, MK, ZA, and KO performed the experiments and analyzed the data. SW, E-KP, and M-KC wrote the manuscript. All authors edited the manuscript and approved the final version.

### Conflict of Interest

The authors declare that the research was conducted in the absence of any commercial or financial relationships that could be construed as a potential conflict of interest.
