# Corrigendum: TRPV1 and TRPV1-Expressing Nociceptors Mediate Orofacial Pain Behaviors in a Mouse Model of Orthodontic Tooth Movement

**DOI:** 10.3389/fphys.2019.01353

**Published:** 2019-10-22

**Authors:** Sheng Wang, Martin Kim, Zayd Ali, Katherine Ong, Eung-Kwon Pae, Man-Kyo Chung

**Affiliations:** ^1^Program in Neuroscience, Center to Advance Chronic Pain Research, Department of Neural and Pain Sciences, School of Dentistry, University of Maryland, Baltimore, MD, United States; ^2^Department of Orthodontic and Pediatric Dentistry, School of Dentistry, University of Maryland, Baltimore, MD, United States

**Keywords:** orthodontic tooth movement, trigeminal ganglia, TRPV1, peptidergic nociceptors, periodontium, behavioral assays

In the original article, there was a mistake in Figure 6 as published. The X-axis of panel B was labeled incorrectly. The corrected Figure 6 appears below.

**Figure 6:**
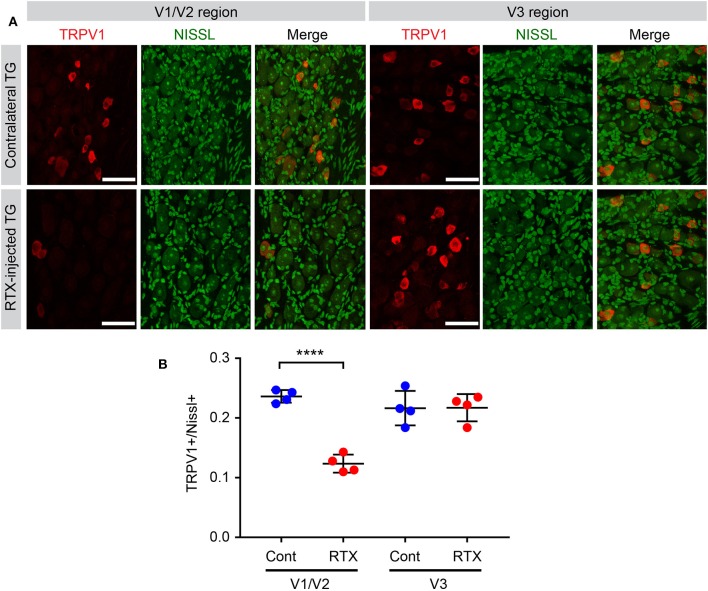
Efficacy of ablation of TRPV1-expressing nociceptors by intra-TG injection of RTX. **(A)** Immunohistochemical labeling of TRPV1 (red), Nissl staining (green), and merged images in ophthalmic/maxillary (V1/V2) area or mandibular (V3) area of TG from RTX-injected or uninjected contralateral side. **(B)** Proportion of TRPV1-expressing neurons among Nissl+ neurons. ^****^p < 0.0001 in Student's *t*-test. *N* = 4 ganglia in each group.

The authors apologize for this error and state that this does not change the scientific conclusions of the article in any way. The original article has been updated.

